# Recovery of smell sense loss by mepolizumab in a patient allergic to Dermatophagoides and affected by chronic rhinosinusitis with nasal polyps

**DOI:** 10.1186/s12948-019-0106-2

**Published:** 2019-02-13

**Authors:** Carlo Cavaliere, Cristoforo Incorvaia, Franco Frati, Daniela Messineo, Mario Ciotti, Antonio Greco, Marco de Vincentiis, Simonetta Masieri

**Affiliations:** 1 Department of Oral and Maxillofacial Sciences, Sapienza University, Piazzale Aldo Moro 5, 00185 Rome, Italy; 2 Cardiac/Pulmonary Rehabilitation, ASST Pini/CTO, Milan, Italy; 3 Private Practice, Camucia, AR Italy; 4 Department of Radiological Sciences, Oncology and Pathology, Sapienza University, Rome, Italy; 5 Department of Sense Organs, Sapienza University, Rome, Italy

**Keywords:** Chronic rhinosinusitis with nasal polyps, Smell sense loss, Eosinophilic, Mepolizumab

## Abstract

**Background:**

Chronic rhinosinusitis with nasal polyps (CRSwNP) frequently presents with dysfunction or loss of the sense of smell, resulting in a significant impairment in quality of life. The medical treatments currently available may improve the olfactory function in patients with CRSwNP, but such an outcome is generally only transitory. We report the case of a patient with CRSwNP who completely recovered from smell sense loss by treatment with mepolizumab.

**Case presentation:**

The patient was a 62-year-old female who has severe asthma induced by allergy to Dermatophagoides and concomitant CRSwNP. Any treatment for the latter, including oral and injective corticosteroids, was unsuccessful in the loss of smell. Due to the satisfaction of admission criteria to mepolizumab treatment for severe asthma, treatment was initiated on March 2018, resulting in good clinical control of both asthma and CRSwNP, and particularly in complete recovery of the smell loss after 4 months of treatment and still persisting.

**Conclusion:**

In this case report, the treatment with mepolizumab in a patient allergic to Dermatophagoides and affected by CRSwNP was associated with an improvement of anosmia. That finding may be explained by a reduction of the nasal obstruction by nasal polyps.

## Background

Chronic rhinosinusitis with nasal polyps (CRSwNP) is characterized by the occurrence for more than 12 weeks of symptoms as nasal discharge, stuffiness, facial pressure or pain, dysfunction or loss of the sense of smell, and cough from post-nasal drip [1]. Among such symptoms, smell loss is strongly associated with impairment in quality of life [2]. The cause of the olfactory loss in patients with CRSwNP is acknowledged in inflammation within the olfactory cleft; the degree of dysfunction is associated with the grade of inflammation and the altered turnover of the olfactory sensory neurons caused by chronic inflammation. The treatment available thus far may significantly improve the olfactory function in patients with CRSwNP, but the improvement is only temporary, and further treatment aimed at persistent elimination of inflammation and promotion of a normal turnover of the olfactory epithelium are warranted [3]. Sinonasal tissue eosinophilia is quite common in CRSwNP, defining the endotype of eosinophilic CRSwNP [4]. Based on the crucial importance of interleukin 5 (IL-5) in promoting eosinophils development and survival, the anti-IL-5 monoclonal antibody mepolizumab is currently evaluated, along with other biologic agents [5], as a treatment for CRSwNP. Anti-IL5 therapy proved beneficial in the treatment of recalcitrant nasal polyposis in selected populations and significantly reduced smell loss starting from 9 weeks of treatment compared with placebo [6]. However, no patient achieving complete recovery of smell sense was reported. We describe the case of a patient allergic to dust mites with severe asthma and CRSwNP who had a smell loss unresponsive to any therapy. The patient underwent treatment with mepolizumab for severe asthma and achieved, along with asthma control, complete sense of smell recovery after 4 months of treatment.

## Case presentation

**Fig. 1:**
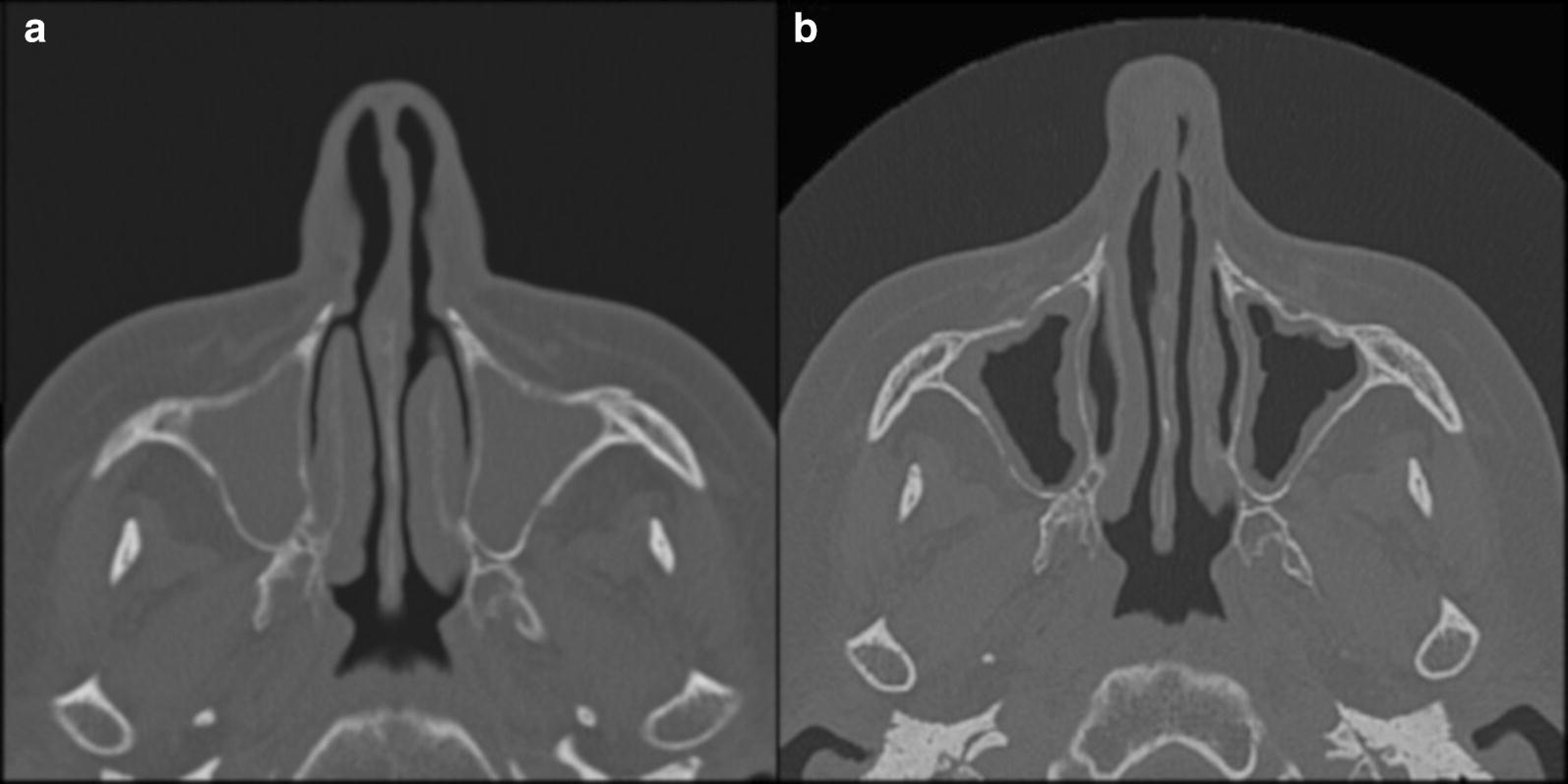
Axial CT comparison before and after therapy: paranasal sinuses CT in axial projection at t0: bilateral engagement of maxillary sinuses (**a**), and at T1 CT: clear improvement in the maxillary sinuses which show only slight mucosal hypertrophy (**b**)

**Fig. 2:**
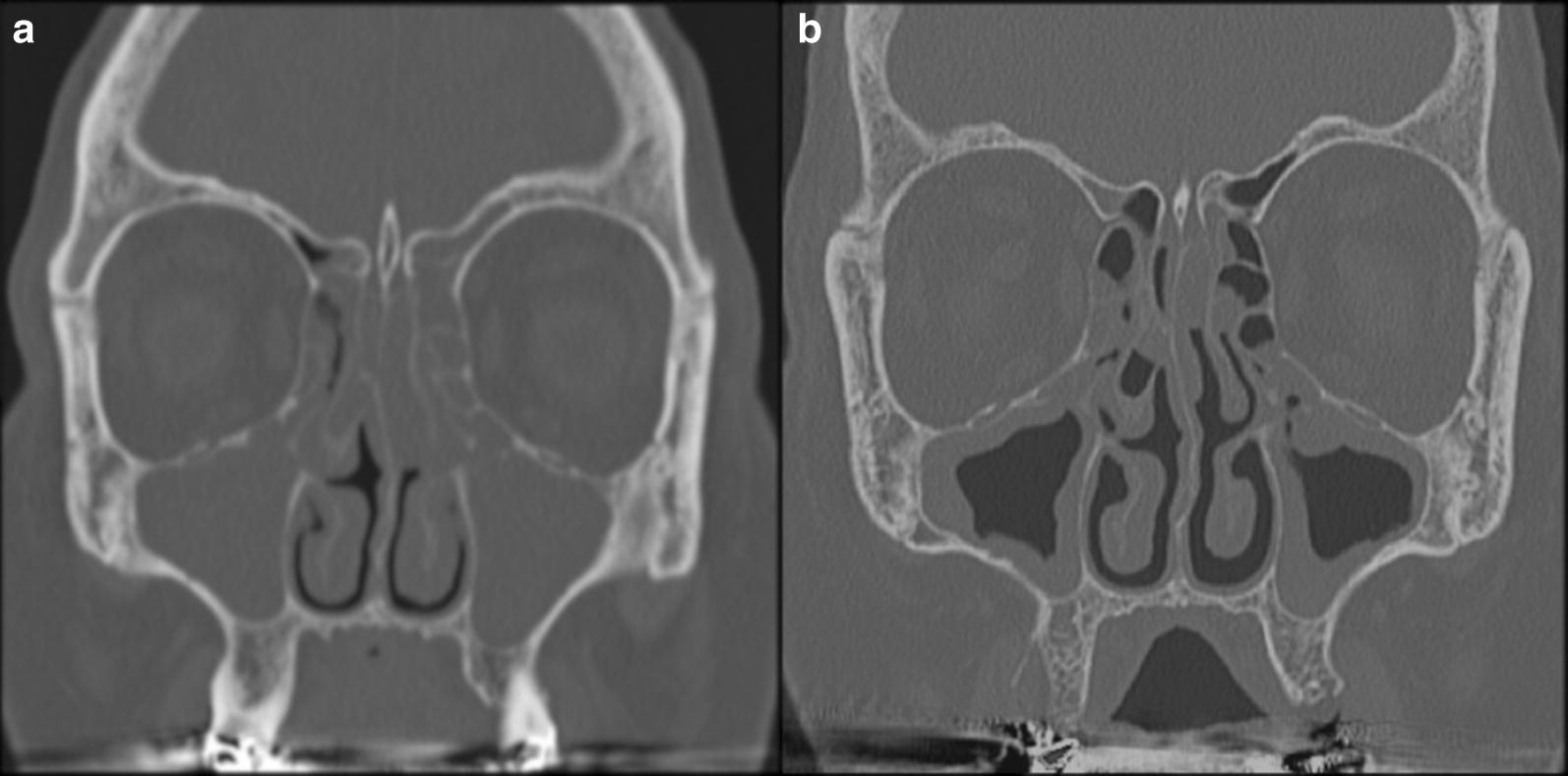
Coronal CT comparison before and after therapy: paranasal sinuses in coronal projection at T0: engagement of both maxillary sinuses, nasal cavities and ethmoid (**a**), and at T1: visible improvement of both maxillary sinuses, with almost complete patency of the nasal cavities and the ethmoid (**b**)

The patient was a 62-year-old female suffering since adolescence from mite-induced asthma, as assessed by positive in vitro testing to *Dermatophagoides pteronyssinus* and *Dermatophagoides farinae*, with development in latest years of severe asthma not controlled by standard drug treatment. The patient fulfilled the admission criteria to mepolizumab treatment, as defined by severe asthma from ≥ 12 months despite high-dose inhaled corticosteroids (ICS) plus additional controller(s) treatment, ≥ 2 exacerbations (requiring systemic corticosteroid and/or ED visit and/or hospitalization in prior 12 months) and blood eosinophil ≥ 150 cells/µl at visit 1 or historically ≥ 300 cells/µl [7]. Lung function measurement by plethysmography showed a forced expiratory volume in 1 s (FEV1) of 64% and significant reversibility to 80% following inhalation of salbutamol 400 µg. From 1998, the patient also suffered concomitant CRSwNP. The disease state was investigated by computed tomography (CT), which showed a picture of pansinusitis with almost complete obliteration of all the paranasal cavities, with erosive reabsorption phenomena associated to the presence of numerous polypoid formations in the ethmoidal cells, extending to the nasopharynx. The patient was previously treated with many drugs, including oral and injective corticosteroids, with some benefit on nasal discharge, stuffiness, facial pressure, and cough but no effect on the loss of the sense of smell. Starting from March 2018, mepolizumab treatment by 100 mg at monthly intervals was performed, that resulted in good clinical control of both asthma and CRSwNP, a complete recovery of the smell loss occurring in the latter after 4 months of treatment and persisting. Figures 1 and 2 show the results of paranasal sinuses CT before (T0) and after (T1) mepolizumab treatment in axial and coronal projection, with evident improvement after treatment.

## Discussion and conclusion

The introduction of biologic therapies for severe asthma was a milestone in the management of this challenging condition. Of interest, biologic agents were found to be successful in several other disorders, as demonstrated for the monoclonal anti-IgE antibody omalizumab, which is effective on a large array of pathologies, even not IgE-mediated. This is true for chronic spontaneous urticaria, for which omalizumab treatment was licensed, and also for CRSwNP, though for such disease omalizumab therapy is off-label [8]. The anti-IL-5 monoclonal antibody mepolizumab is indicated in the severe eosinophilic asthma phenotype. In a cohort of 670 patients with severe asthma candidate for biologic treatment, 20% were eligible for mepolizumab [7], and the studies available thus far demonstrated the effectiveness of this treatment.

Similarly to asthma, also CRSwNP comprises an eosinophilic endotype, and the meta-analysis of five studies (four randomized control trials (RCT), one case–control and two case series) investigating the treatment outcome, demonstrated a standard mean difference of improvement in nasal polyposis significantly higher for both omalizumab and mepolizumab compared with placebo. In particular, anti-IL5 treatment resulted in a reduction in nasal polyp score. These data suggested the authors conclude that biologic therapies may prove beneficial in the treatment of recalcitrant nasal polyposis in selected populations [5]. To achieve complete recovery of smell loss was not considered thus far, due to the unsatisfactory results obtained by all previous treatments, as a credible need to meet. In the case reported, the treatment with mepolizumab was associated with a marked improvement in the olfactory capacity of the patient. Also, the other symptoms of CRS regressed, even if, unfortunately, we did not objectivize it by assessing a clinical score as the 22 items Sino-Nasal Score (SNOT-22). Although the results obtained in our patient were promising, it should be remembered that the patient was allergic to Dermatophagoides and therefore it would not be correct to extend our observation to patients allergic to other types of allergens.

The case we have observed proposes to perform specific studies on patients with CRSwNP who have developed anosmia to verify the ability of mepolizumab to work on a symptom significantly impairing the patient’s quality of life.

## Abbreviations

CRS: chronic rhinosinusitis
CRSwNP: chronic rhinosinusitis with nasal polyps
IL-5: interleukin 5
ICS: inhaled corticosteroids
FEV1: forced expiratory volume in 1 s
CT: computed tomography
